# Navigating Medicine’s Uncertainties

**DOI:** 10.7759/cureus.102415

**Published:** 2026-01-27

**Authors:** David W Frost

**Affiliations:** 1 General Internal Medicine, University of Toronto, Toronto, CAN

**Keywords:** ambiguity, diagnostic reasoning, medical education, psychology, uncertainty

## Abstract

This editorial article is grounded in the bedside experience of uncertainty in medicine. It is a narrative, conceptual synthesis drawing extensively on evidence rather than a systematic review. It frames the ubiquity and relevance of uncertainty in medicine, summarizing some existing concepts and frameworks, also drawing from non-medical fields. Ambiguity is distinguished as a subtype of uncertainty, and some of what is known about the subjective experience of uncertainty is presented. This editorial summarizes some of the literature on communicating uncertainty effectively and explores the link between uncertainty and diagnostic reasoning and cognitive biases. It concludes with suggestions and practical tips for normalizing and grappling with this constant in our work lives.

## Editorial

It takes little time assessing, diagnosing, and treating patients to realize the ubiquity of uncertainty - be it diagnostic uncertainty, the uncertainty relating to diagnostic test interpretation, the experience of patients and their families, or myriad other types. It touches all aspects of care, and busy clinicians spend much their professional lives navigating the uncertainty experienced by themselves and their clinical colleagues, by their patients and their families, and, in a teaching setting, by the learners in their charge. Yet, despite increasing attention to the topic in the medical literature and lay press [1], clinicians report feeling isolated, inadequate, or threatened by uncertainty [2,3]. Despite calls and proposals to address the topic in medical education [4-6], approach to uncertainty seldom appears in medical school and residency curricula [7] and is typically learned through experience and discussions with trusted colleagues and mentors. There is a wealth of knowledge to be gleaned from both medical and non-medical fields on classifying, acknowledging, and demystifying uncertainty, which can lead to a more satisfying and less isolating clinical practice and truly shared decision-making with patients and their families.

In this editorial, I summarize concepts and frameworks, distinguish ambiguity as a subtype of uncertainty, present some of what is known about the subjective experience of uncertainty, summarize some of the literature on communicating it effectively, and conclude with suggestions and tips for normalizing this constant in our work lives.

Concepts and frameworks of uncertainty in medicine

Psychologist Michael Smithson created a well-known “Taxonomy of Ignorance” [8]. In this paradigm, uncertainty is a subtype of ignorance stemming from incomplete information, which can be further subdivided into probability and ambiguity. Probability refers to situations where the parameters and possible outcomes are generally known, but not the specific outcome in a given case. A dice or card game are non-medical examples. In medicine, probability may manifest as the question of whether a patient will respond to a therapy. Ambiguity refers to a situation where the parameters or possible outcomes are unknown and may be reducible with more or better information. Non-medical examples include a dice with unknown number of sides or numbers, or a non-standard deck of cards. A medical example is a more nebulous question like “does this patient with weight loss and mild anemia have a serious medical problem, and, if so, what is it?”

A related but different distinction between subsets of uncertainty is aleatoric and epistemic. Aleatoric uncertainty refers to randomness and chance and is irreducible; “necessary uncertainty” and “unknowable unknowns” are descriptors. Clinical examples include “will this person with an extensive smoking history develop lung cancer?” and “will this patient have an adverse effect from this medication?” Epistemic uncertainty refers to incomplete knowledge and is potentially reducible with more or better knowledge. Descriptors include “unnecessary uncertainty” and “knowable unknowns.” Clinical examples include “is the pathologic diagnosis correct?” and “does this center employ the best possible measures to prevent drug administration errors?” Aleatoric and epistemic uncertainty are not immutable; concepts could move from aleatoric to epistemic as more is learned; what seemed random at an earlier point may prove not to be random once previously unknown mechanisms or processes are elucidated. For example, allopurinol hypersensitivity syndrome, once thought an unpredictable random drug reaction, is now known to be associated with a nearly 100-fold increased risk in individuals with HLA-B*58:01 [9]

Several comprehensive frameworks for uncertainty in medicine have been developed. A famous model framed by the sociologist Renee Fox [10] divides uncertainty into three categories: 1) uncertainty stemming from individual awareness of aspects of medicine that are known, 2) uncertainty stemming from the medical profession’s ignorance of a condition, and 3) inability to distinguish between 1 and 2.

The uncommon, recently described VEXAS syndrome is illustrative of the profession’s collective ignorance. In 2020, Beck et al. [11] described 25 patients with somatic mutations in the enzyme UBA1 and coined “VEXAS syndrome” (Vacuoles, E1-enzyme, X-linked, Autoinflammatory, Somatic). Prior to the description of this syndrome, many patients had met criteria for various other inflammatory disorders such as relapsing polychondritis, polyarteritis nodosa, giant cell arteritis, or a hematologic condition. How many patients with “idiopathic,” unknown, or diagnoses considered certain have a similar situation? This framing can be helpful in counselling patients with medically unexplained physical symptoms.

In a more recently published framework focused on the journey of a patient with cancer, Paul Han, oncologist and scholar in medical uncertainty, divides uncertainty into the categories of scientific, healthcare system-related, and personal [12]. This is an attractive framework as it illustrates the ubiquity and complexity of uncertainty in what appears to be a routine clinical problem across multiple stages of an illness.

 How uncertainty permeates medical decision-making

The notion of constantly attempting to eliminate uncertainty is deeply engrained in medical culture and has been termed an “ideology of uncertainty reduction” [12]. Seeking the most parsimonious unifying diagnosis - Occam’s Razor - is taught early and often. Perhaps less known is Hickam’s dictum: “patients can have as many diseases as they damn well please” [13]. There is often an emphasis on labelling and categorizing presentations and committing to a diagnosis. Each of these concepts has a place in diagnostic reasoning and can indeed often be positive. However, over-emphasizing this at the exclusion of openness and acknowledgment of uncertainty may lead to well-known cognitive errors shown in Table 1 [14].

**Table 1:** Heuristics and biases that may lead to excessive certainty

Heuristics and biases
Anchoring
Blind spot
Confirmation
Framing
Groupthink
Overconfidence
Salience
Stereotyping

Uncertainty has positive aspects: it drives research and innovation, stimulates intervention, and may lead to a positive cascade as answers beget ever more detailed and sophisticated questions. Acknowledging limitations also leads to a humbler approach to medicine and may counter a formulaic approach to patient care.

Clinicians constantly make maneuvers to mitigate or reduce uncertainty. These include obtaining more data (be it through a more detailed history and physical or, more commonly, through more testing), requesting a consultation, changing the setting of care, arranging closer follow-up, conducting a therapeutic trial, or deferring and allowing the “test of time.” However, as effective as these maneuvers usually are, they are not perfect. More data, for example, do not always lead to more certainty. Incidental findings can arise from ordering imaging studies. Distinguishing signal from noise in any panel of investigations can be challenging. Ordering nonspecific tests (e.g., inflammatory markers) may lead to no more certainty and reminds clinicians to consider what they would do with a given result before ordering a test.

**Figure 1:**
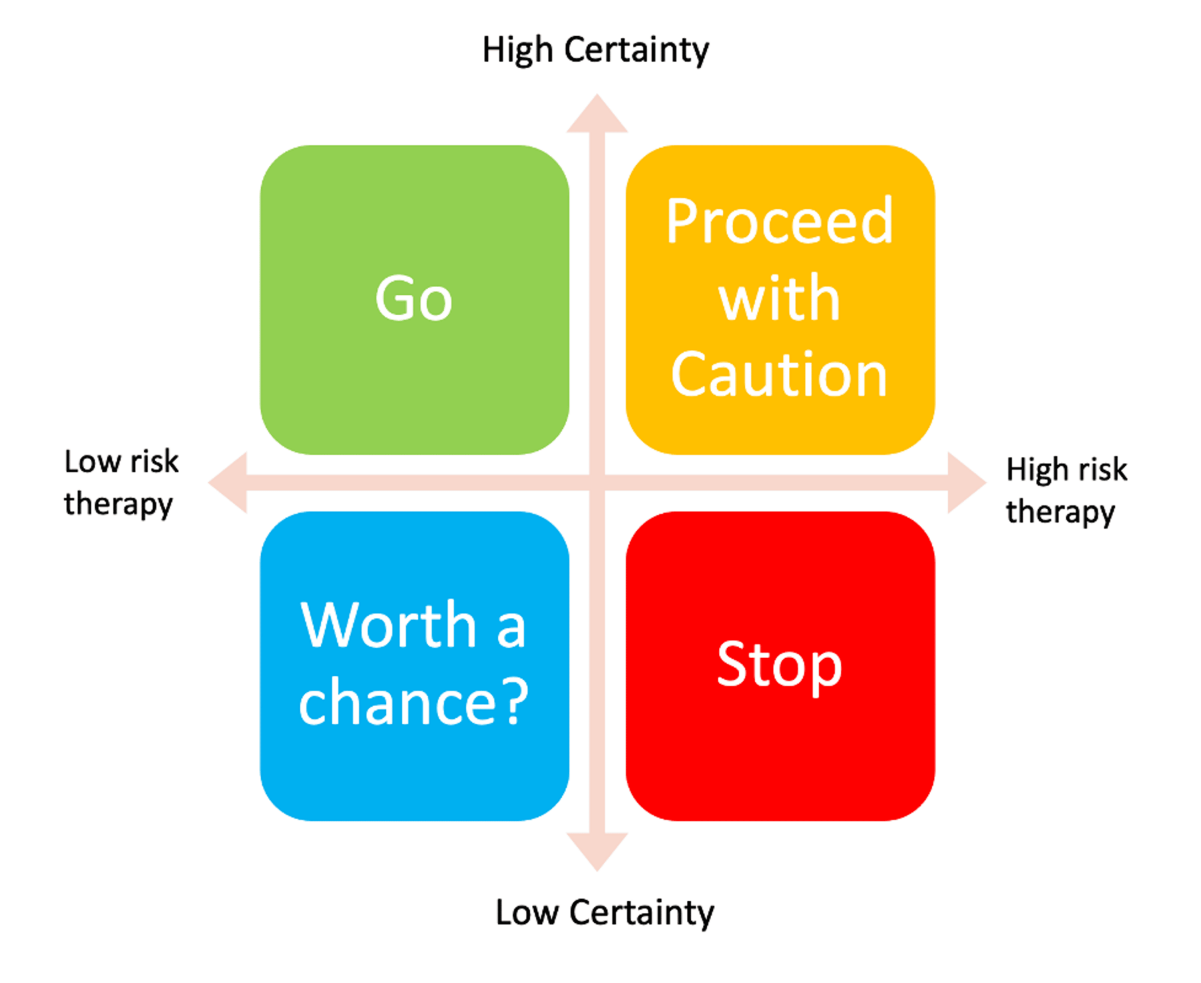
Diagnostic certainty and risk matrix Original figure created by the author

Information that is sometimes treated as absolute often remains subject to uncertainty. Pathology or radiology results may fall into this category. However, a clinician who never accepts any degree of certainty as sufficient will quickly become paralyzed and unable to function. A more pragmatic approach is the concept of “epistemic threshold” - regarding certainty as a threshold to inform behavior rather than as an absolute. The following quotation from Kassirer summarizes the notion: “Our task is not to attain certainty, but rather to reduce the level of diagnostic uncertainty enough to make optimal therapeutic decisions” [15]. The degree of certainty required before initiating action depends on factors including the risk or toxicity of the proposed therapy, the risk of the untreated condition in question, and the risk or invasiveness of attempting to obtain more certainty. Some of this is illustrated in the matrix shown in Figure 1.

Subjective, psychological, and physiological experience of uncertainty 

With the caveat that experiments do not necessarily simulate clinical reality, several lines of evidence suggest that uncertainty is highly aversive, including studies of physiologic stress in both humans and other animals [16]. A notable study, examining at humans’ physiologic response to uncertainty, found that the uncertain state produced maximal physiologic stress response, even more so than certainty of an adverse outcome - in this case an electric shock [17].

A series of experiments by the American economist and military strategist Daniel Ellsberg in the 1960s [18] demonstrated what has been termed the “Ellsberg Paradox”- that most people will choose a condition of uncertainty with known parameters over a more ambiguous condition with some unknown parameters, even when there is no mathematical advantage to the unambiguous condition. This has been described as “ambiguity aversion.” Though the details of these experiments are outside the scope of this article, interested readers may find the many written or video descriptions of Ellsberg’s “Urn experiments” illuminating [19]. There may be implications in medicine where, as outlined above, within uncertainty, some situations are more ambiguous (epistemic uncertainty) and some have more defined parameters (aleatoric uncertainty).

Patient experience and communicating uncertainty

Clinicians may be faced with possible tension between respecting patient autonomy though transparent disclosure of uncertainty and maintaining their confidence. There are some strategies that may assist in this regard. A clinical scenario-based study examined three different ways of expressing uncertainty and found that implicit communication of uncertainty, through a differential diagnosis or through expressing probabilities, engendered more confidence than an explicit declaration of uncertainty [20]. This way of expressing uncertainty accurately reflects clinical practice, where we are rarely at a complete loss for an explanation but are rather simultaneously working though different diagnostic possibilities.

The National Academy of Medicine’s document “Improving Diagnosis in Healthcare” [21] recommends sharing a working diagnosis including degree of certainty and when and whom to contact if there is no resolution or symptoms arise that do not fit the working diagnosis. The caveat is that a large proportion of patients will be unfamiliar with the concept of a differential or working diagnosis, and the potential role for watchful waiting, and may require education. Other strategies that can be useful in communication include pre-warning prior to initiating a diagnostic workup, e.g., “we may not find a precise diagnosis but we will try to rule out serious causes.”

Conclusions and recommendations

Uncertainty is inevitable and ubiquitous in medicine. Efforts have been made to subclassify uncertainty, as not all uncertainty is the same. Any uncertainty, perhaps particularly ambiguity, is aversive to humans - patients and providers alike - making its navigation particularly challenging. In seeking accurate diagnoses, clinicians need to navigate the balance of obtaining enough, but not excessive, certainty. This is not straightforward, and clinical humility and honesty about the degree of certainty both with oneself and one’s colleagues, as well as patients and their families, may decrease diagnostic error in addition to providing the transparency needed to practice ethically.

Notwithstanding the complexity of the topic, there are measures clinicians can consider taking to address the various challenges discussed here. One suggestion is to normalize uncertainty and become comfortable communicating it. Another suggestion is to strive to be aware of situations where our inherent drive to avoid uncertainty as clinicians risks diagnostic error. It can be useful to think of certainty as a threshold to action rather than absolute. In communicating with patients, most appreciate communication of uncertainty, perhaps to a greater extent than we think, but how best to do this while maintaining confidence is not straightforward and requires care. Finally, acknowledging and sharing our uncertainty with our colleagues and our patients may make practicing medicine more satisfying and less isolating and lead to a more genuine and honest interaction with our patients.
